# Current News

**Published:** 1900-11

**Authors:** 


### Current News.

### DENTAL COMMISSIONERS OF CONNECTICUT.

The Dental Commissioners of Connecticut will meet at the Capitol in Hartford, Tuesday, Wednesday, and Thursday, November 13, 14, and 15, 1900, to examine candidates for license and attend to any business proper to come before them.

The written theoretic examination Tuesday and Wednesday, November 13 and 14.

Practical examination in operative and prosthetic dentistry at nine o'clock, Thursday, November 15.

All persons desiring to practise dentistry in this State must apply to the Recorder for *revised rules* and for the proper blanks. Blanks must be carefully filled in and sworn to and with the fee, twenty-five dollars ($25.00), filed with the Recorder at least one week before the day of examination.

GEO. L. PARMELE, M.D., D.M.D.,
*Dental Commissioner and Recorder.*

HARTFORD, September 15, 1900.

---

### INSTITUTE OF DENTAL PEDAGOGICS.

THE seventh meeting of the Institute of Dental Pedagogics will be held in Nashville, at the Maxwell House, begining at ten o'clock, Thursday, December 27, 1900, and will continue three days.

The programme will be forwarded to all the journals in time for publication in the December issues. Every one interested in Dental teaching should feel it his duty to attend, as it is his privilege to speak on any subject on the programme.

The interest of last year will be maintained, and the plan of developing thoroughly a few topics given a trial.

A most cordial invitation is extended to all, especially to those who are teachers.

HENRY W. MORGAN,
D. M. CATTELL,
W. E. WILMOTT,
*Executive Committee.*

